# Virtual Reflection Group Meetings as a Structured Active Learning Method to Enhance Perceived Competence in Critical Care: Focus Group Interviews With Advanced Practice Nursing Students

**DOI:** 10.2196/42512

**Published:** 2023-03-23

**Authors:** Marianne Trygg Solberg, Anne Lene Sørensen, Sara Clarke, Andrea Aparecida Goncalves Nes

**Affiliations:** 1 Lovisenberg Diaconal University College Oslo Norway

**Keywords:** advanced practice nurse, nursing education, virtual reflection group, teaching design, critical care, active learning approach

## Abstract

**Background:**

Advanced practice nurses (APNs) are in high demand in critical care units. In Norway, APNs are educated at the master’s degree level and acquire the competence to ensure the independent, safe, and effective treatment of patients in constantly and rapidly changing health situations. APNs’ competence embraces expert knowledge and skills to perform complex decision-making in the clinical context; therefore, it is essential that educational institutions in nursing facilitate learning activities that ensure and improve students’ achievement of the required competence. In clinical practice studies of APN education, face-to-face reflection group (FFRG) meetings, held on campus with the participation of a nurse educator and advanced practice nursing students (APNSs), are a common learning activity to improve the competence of APNSs. Although FFRG meetings stimulate APNSs’ development of required competencies, they may also result in unproductive academic discussions, reduce the time that APNSs spend in clinical practice, and make it impossible for nurse preceptors (NPs) to attend the meetings, which are all challenges that need to be addressed.

**Objective:**

This study aimed to address the challenges experienced in FFRG meetings by implementing virtual reflection group (VRG) meetings and to explore the experiences of APNSs, NPs, and nurse educators in VRG meetings as an active learning method supported by technology to stimulate students’ development of the required competence to become APNs in critical care.

**Methods:**

This study adopted a qualitative explorative design with 2 focus group interviews and used inductive content analysis to explore the collected data.

**Results:**

The main finding is that reflection group meetings supported by technology resulted in a better-structured active learning method. The VRG meeting design allowed APNSs to spend more time in clinical practice placements. The APNSs and NPs experienced that they participated actively and effectively in the meetings, which led to a perceived increase in competence. The APNSs also perceived an improved learning experience compared with their prior expectations.

**Conclusions:**

Users perceived that the implemented novel teaching design supported by technology, the VRG meeting, was a more effective method than FFRG meetings on campus to develop APNSs’ required competence in critical care. The VRG was also perceived as an improved method to solve the challenges encountered in FFRG meetings. Specifically, the APNSs felt that they were prepared to undertake complex decision-making with a higher level of analytic cognition in a clinical context and to lead professional discussions in the ward. This developed teaching design can easily be adapted to diverse educational programs at various levels of professional education.

## Introduction

### Overview

Worldwide, health care institutions’ treatment of patients has become increasingly complex because of the rapidly aging population [1,2]. In addition, treatment and technological developments allow chronically ill patients to manage their diseases at home longer than before so that when they need to be treated in health care institutions, their health situation is worse and more complex than that of patients a few years ago [3]. The recent global COVID-19 pandemic presented an unexpected situation in which many infected persons required acute critical care, but knowledge of treatment was scarce, creating an urgent desire for the ability to address the situation rapidly and critically [4]. These developments highlight the need to prepare advanced practice nurses (APNs) in their education to face contemporary challenges in critical care. The role of an APN requires expert knowledge and skills to make complex decisions in a clinical context [1].

To become an APN in Norway, registered nurses must attend and complete a master’s program of 120 European Credit Transfer and Accumulation System points. The curriculum is designed to guide an advanced practice nursing student (APNS) to acquire the expected competence. To educate APNs in critical care, it is essential to offer learning activities that promote the development of professional competence needed to care for acutely or critically ill patients. The main competencies that APNSs must acquire in their education are biophysical knowledge, technical skills, communication skills, intra- and interprofessional teamwork skills, leadership skills, and guidance and coaching skills as well as knowledge of evidence-based practice [5,6]. Furthermore, it is essential for APNSs to develop core qualities and competencies for patient safety [5,7]. Overall, nurses’ greater educational qualifications are associated with better patient outcomes [8]. APNs are in great demand in critical care units (CCUs) because they can ensure independent, safe, and effective practices in constantly and rapidly changing situations [1,2,5,9].

### Background

The APN master’s program at Norway’s Lovisenberg Diaconal University College (LDUC) provides theoretical and clinical practice studies over a period of 2 years. The clinical practice studies are distributed over a period of 8 weeks in the first term, 12 weeks in the second term, and 9 weeks in the third term. In each term, APNSs study various theoretical subjects before and after their practical period. In the last term, they focus on their master’s thesis.

A crucial part of nursing education is helping students to develop a strong foundation of evidence-based practice skills and apply them in clinical practice [10]. Therefore, the LDUC’s advanced practice nursing master’s program in critical care uses reflection group (RG) meetings as a learning activity. The RG meetings aim to train students to reflect on their experiences during the clinical practicum period, supporting their reflections with evidence. In this process, called *reflective practice*, students critically consider and assess their practical experiences to gain knowledge and learn how to improve their competencies and skills [11,12]. Analyzing clinical problems in evidence-based practice through critical reflection demands combining the best available research evidence, expert opinions, and patients’ individual preferences [13]. Nurses who learn to reflect on their practical experiences develop professional competence to solve problems and provide more flexible, individualized, and holistic care to patients [14].

RG meetings at LDUC have recently been held on campus, with APNSs participating in 3 group sessions of 3 hours in each practicum. During the meetings, which included up to 10 APNSs, each student presented a patient case from clinical practice and a related research paper, providing evidence as recommended by Straus et al [13]. RG meetings aim to stimulate reflection and facilitate APNSs’ achievement of their expected competence. The structure of face-to-face RGs (FFRGs) led to several challenges, however, including reduced time in clinical practice placements (as the APNSs had to meet the nurse educator [NE] and fellow students at LDUC campus), unproductive professional discussions (as the APNS were often unprepared for meetings), the impossibility of involving nurse preceptors (NPs) in organized professional discussions (as they could not leave the clinical practice for a long period because of their responsibility for patient care), and a perceived low achievement of learning outcomes, as underlined in the assessment meeting of APNSs in clinical practice. In addition, the FFRG format of each student giving a short presentation often leads to repetition in academic discussions.

To address these challenges, the FFRG meeting concept was redesigned according to the LDUC’s strategy of using active learning methods based on technology. The new design, called the virtual RG (VRG) meeting, was better structured and held remotely via the Zoom meeting platform rather than in person. The first course to use the VRG meeting design was the Management of Complex Patient Conditions, the main learning outcome of which was analyzing and managing complex clinical situations based on professional experience, research, and knowledge. APNSs must gradually develop situational awareness and action skills in complex patient situations. Specifically, they must collaborate with the preceptor to gradually act independently using evidence-based practice. The VRG meeting aimed to increase the students’ time in clinical practice placements, to better organize professional discussion and reflection, to optimize and facilitate the participation of the students’ preceptors in RG meetings, and to improve the APNSs’ achievement of expected learning outcomes [15]. Constructive alignment [16] was applied as a theoretical approach in developing the new design, ensuring a connection between learning outcomes, learning activities, and the assessment of clinical practice [15].

Throughout the clinical practice period, the NE, NPs, and APNSs attended VRG meetings, which comprised three meetings of 45-minutes each that were executed over 3 days (see Table 1). Before the meetings, APNSs were assigned roles with specific tasks and responsibilities, giving them time to prepare. The roles were distributed as follows in each meeting session: 1 APNS assumed the role of “responsible student,” another was the “respondent,” and the remainder were ordinary “participants.” The responsible student’s role was to prepare a session of 45 minutes in collaboration with their NP by choosing a patient case and related research paper. The respondent APNS was responsible for critically assessing the research paper, and the remaining students were responsible for being prepared for the meeting by reading the case and research paper. It was also expected that during the discussion, the remaining students actively participated by reflecting on and sharing their own experiences with similar cases with their peers. A structured approach to RG meetings with the participation of an experienced NP can enable nursing students to reach a deeper level of assessment and a higher level of cognition [12,14]. Table 1 provides an overview of the main differences between the FFRG and the VRG.

**Table 1:** Comparison of face-to-face and virtual reflection group meeting design.

	FFRG^a^ meeting design	Consequences of an FFRG meeting	VRG^b^ meeting design	Consequences of a VRG meeting
Attendees	1NE^c^, 9 APNSs^d^	Lack of expert opinions from NPs^e^ in the discussions	1 NE, 1 NP, 9 APNSs	Included expert opinions from NPs in the discussions
Setup and location	3 FFRG meetings of 3 hours each at the university college campus Led by the NE Time per APNS presentation and discussion in the meeting: 15 minutes	The APNSs spent a total of 9 hours away from the clinical practice placement. Travel time was needed. NPs were not able to join the RG^f^, as travel and discussion would require too much time away from critically ill patients.	3 VRG meeting sessions of 45 minutes, totaling of 2 hours and 15 minutes on each Zoom meeting Each session was led by 1 APNS. Time per APNS presentation and discussion in each session: 45 minutes	The APNS spent a total of 6 hours and 45 minutes away from the clinical practice placement. No travel time was needed. The NPs were able to join the professional discussions, as they could join the meetings in a room close to critically ill patients.
Content	9 APNSs each presented a patient case from clinical practice placement and a research article related to the case. The presentation was followed by a group discussion.	Various levels of APNSs’ preparation for the participation Short presentations by all APNSs, often leading to repetition in the professional discussions	3 APNSs each presented a patient case from clinical practice placement and a research article related to the case. The patient case was sent to the participants before the meeting. The presentation was followed by a group discussion.	The APNSs were prepared for participation. Long presentations by APNSs, allowing time for thorough professional discussions
Tools	None	Unstructured discussion	Zoom video conferencing platform	Structured discussion and participation order based on raised hands.
Participants’ roles	The NE was responsible for the discussion section. The role of APNS respondent was not defined. APNSs had no defined responsibilities.	Passive participation of APNSs	An APNS was responsible for the organization of the discussion section. An APNS respondent critically assessed the chosen article in advance and presented the assessment to the group at the meeting. Constructive participation in the discussion section was expected from the APNSs.	Active participation of APNSs

^a^FFRG: face-to-face reflection group.

^b^VRG: virtual reflection group.

^c^NE: nurse educator.

^d^APNS: advanced practice nursing student.

^e^NP: nurse preceptor.

^f^RG: reflection group.

### Objectives

This study aims to address the challenges experienced in FFRG meetings by implementing VRG meetings and to explore the experiences of APNSs, NPs, and NEs with VRG meetings as an active learning method to stimulate students’ development of the competencies needed to become APN in critical care.

### Research Questions

The research questions are as follows:

1. How did VRG meetings address the challenges experienced in FFRG meetings?
2. In comparison with FFRG meetings, what were the experiences of APNSs, NPs, and NEs with VRG meetings as an active learning method to stimulate the students’ perceived development of the competencies needed to become an APN in critical care?

## Methods

### Design

This study adopted a qualitative explorative design using focus group interviews. An exploratory design is useful for identifying views and experiences [17] regarding, in this setting, users (NSs, NPs, and NEs) participation in VRG meetings.

### Participants

The participants recruited for this study were APNSs, NPs (from the CCUs where the students carried out their clinical practice), and NEs from the master’s program in APN in critical care. The APNSs were recruited from 2 cohorts (autumn 2018 and autumn 2019). To be eligible for the study, the participants (APNSs and NEs) had to have had experience with the previous FFRG meeting design before participating in the VRG meetings. The course coordinator and associate professor (MTS) and the assistant professor (Ørjan Flygt Landfald) were responsible for the concept and organization of the VRG meetings.

### Data Collection

Information about the study and invitations to participate were disseminated to the APNSs and NEs via the Canvas (Instructure, Inc) learning platform. The NPs were contacted via email because they had no access to Canvas. Informational meetings were also arranged after the users’ participation in the VRG meetings to recruit informants for the focus group interview. Polit and Beck [17] recommend that participants should feel no pressure to participate in research studies, so those interested in participating in focus group interviews had to contact the researcher (Ørjan Flygt Landfald). The researcher (Ørjan Flygt Landfald) had no previous contact with the APNSs or NPs, thus avoiding a potential influence on the recruitment of participants or the content of the collected data.

### Data Generation and Setting

To inform the focus group interviews, the research team developed an interview guide with open-ended questions about participants’ (APNSs, NPs, and NEs) experiences with the VRG (Textbox 1). The research team was trained in advance to conduct the interviews. A total of 2 focus group interviews were conducted immediately after the students’ clinical practice periods: the first in October 2019 (third semester) and the second in April 2020 (second semester). The first focus group interview was held in a conference room on the LDUC campus. Participants were seated around a table to indicate an equal position in the discussion. The second focus group was web based because of the COVID-19 pandemic.

**The interview guide.**

- Main question
  Can you talk about your experiences of participating in virtual reflection group (VRG) meetings as compared with the face-to-face reflection group meetings?
- Supporting questions
  What are the benefits and limitations of the VRG meetings?
  What was your experience of following a guide for conducting VRG meetings?
  What competencies did you develop from the VRG meetings regarding your role as an advanced practice nurse (APN)?
  How did the professional discussion contribute to your development as an APN in critical care?
- Different roles are included in the implementation of a VRG; what expectations did you have in advance regarding:
  leading the professional discussion when conducting a VRG?
  including the nurse preceptor in the discussion to share their experiences?
  your role as a respondent?

Research shows that the manner in which an interview is conducted crucially determines the quality of the collected data and relies on the moderator’s proficiency [17]. The moderator in this study (AAGN) was an experienced researcher with a PhD. In the interviews, 2 members of the research group were observers (Irene Rød and Ørjan Flygt Landfald) and were allowed to make comments and follow-up questions if they perceived a need for them. In the first interview, Irene Rød observed and took notes on the group’s interactions to supplement the verbal transcript and enable a fuller analysis, as recommended by Polit and Beck [17]. In the second interview, Ørjan Flygt Landfald participated and organized technical support and audio file recording. The moderator was familiar with the required competencies of APNSs and encouraged the informants to actively participate in the conversation. The participants freely commented on each other’s views and experiences of VRG meetings as an active and effective teaching method. The focus group sessions lasted 60 minutes and were audiotaped and subsequently transcribed verbatim by MTS using the HyperTRANSCRIBE tool.

### Ethics Approval

The project was approved by the Norwegian Center for Research Data (reference number: 132520). After the participants (APNSs, NPs, and NEs) expressed interest in participating, the course coordinator and associate professor (MTS) again provided verbal and written information about the study, after which the participants provided written informed consent. Before signing the informed consent, they were made aware that participation in the study was voluntary, that they could withdraw their consent at any time without giving a reason, and that doing so would not affect their study conditions at LDUC. The NPs and NEs were assured that their participation in the study would not affect their work conditions. The collected data were treated confidentially and used as described for the purpose of the project. The data were anonymized, making it impossible to identify individuals.

### Data Analysis

All authors participated in the data analysis, first reading the transcripts several times to gain insight into the content. We used inductive content analysis as described by Graneheim and Lundman [18] to explore APNSs’ perceived achievement of the required competence in critical care after participating in VRG meetings. Next, the text was condensed into meaning units with descriptions close to the text, and codes were inductively developed by reading and rereading the meaning units. We had several discussions and finally agreed on categorizing the results into subthemes and themes (Table 2). During the analysis, we moved forward and backward between themes and subthemes, as recommended in the literature [18,19].

**Table 2:** Examples of the analysis process from meaning unit to theme.

Raw data divided into meaning units	Condensed meaning unit description close to the text	Interpretation of the underlying meaning	Subtheme	Theme
NP^a^ 5: We had a difficult case that the student chose to take up, which involved several people in the unit, so we talked. Both I and the student and several others also talked about the case before she presented it [in the VRG^b^ meeting]. And after the VRG, it also was talked about, because it was a case that many thought was a bit difficult, and it became a learning situation for the students, of course, but also for the colleagues in the unit. So, it was actually quite a useful method and there were more people who benefited by learning from it then.	The preceptor described that the student chose to discuss a difficult case in the VRG meeting that led to a great involvement of colleagues working in the CCU^c^. Both APNSs^d^ and colleagues on the unit learned from the discussions.	The VRG meeting is organized in a way that led to a great deal of involvement and increased the competence of students, the preceptor, and employees in the CCU.	Improved focus on evidence-based practice in the clinical environment	Synergy in competence development
Moderator: Several of you have said that it is scary. What is scary? APNS 5: It was probably what was supposed to happen, because you shall lead the meeting for an entire hour, which none of us has done before. You will welcome, then you will present a case, then you will present an article, then you have questions [the fellow students] what do you think...and what do you think? You have to “hold it all the time.” Then, it’s not as simple as someone has mentioned earlier here, that you just talk as you can in a normal discussion, but that you actually have to “name drop” the other students as I did. If no one is talking, then I “name drop” in a way [laughs] so that there will not be such silence. So, yes, there was a bit of that about being a leader, which was scary, but it was very educational.	The APNS thought it was both scary and educational to be responsible for leading the meeting for 45 minutes the first time. Their charge was to welcome the participants, present a case and an article, and include all the students and preceptor in the discussion so that they all actively contributed to the discussion instead of participating in silence.	The role of leading a meeting and presenting a case and research article while making sure that all the students participated was quite scary the first time but at same time very educational.	Increased leadership skills	Developed intrapersonal and professional skills

^a^NP: nurse preceptor.

^b^VRG: virtual reflection group.

^c^CCU: critical care unit.

^d^APNS: advanced practice nurse student.

### Trustworthiness

Participants (APNSs, NPs, and NEs) were divided into 2 groups. Each group was interviewed by the moderator, who was an associate professor (AAGN) who led the group discussions according to the prepared guide. Moderator bias was minimized, as both the moderator and observer were not involved in teaching the APNSs and were completely unknown to the participants [17]. Data saturation was achieved in the second interview, as no new information was obtained and redundancy of the collected data was achieved. These findings reflect a deep understanding of the data because of the authors’ diverse areas of expertise. MTS is an associate professor, is a coordinator of APN master’s education, and has experience in critical care; AAGN is an associate professor in nursing undergraduate education with clinical experience with chronically ill patients; SC is an assistant professor and was the head LDUC librarian; and ALS is an associate professor, is the head of the master’s department at LDUC, has for several years completed training in practice guidance, and has clinical experience in hospital and nursing home medical departments. In addition, 3 of the authors had extensive research experience with qualitative design and data analysis. All the authors have agreed on the final results.

The NEs were responsible for delivering the intervention through VRG meetings but were not involved in student recruitment or data collection. The researchers responsible for data collection had no previous contact with the APNSs to avoid possible bias connected to students being afraid that positive or negative feedback in the interviews could influence their grades.

## Results

### Overview

The eligible participants comprised approximately 35 APNSs, 10 NPs, and 3 NEs who participated in VRG meetings as a part of learning activities in advanced clinical practice (Table 3).

To ensure anonymity, references to individual participants’ statements used nonidentifying numbers to represent the individuals (Table 4).

**Table 3:** Participants in the study.

	Focus group 1 (in person), participated/invited	Focus group 2 (web-based), participated/invited	Total, participated/invited
Students	6/8	3/27	9/35
Preceptors	4/5	2/5	6/10
Educators	2/2	2/2^a^	4/4
Total	12/15	7/34 (−1)^a^	19/49 (−1)^a^

^a^One educator guided the groups in 2 different clinical practice periods; therefore, this educator participated in both focus group interviews 1 and 2.

**Table 4:** Overview of participants’ nonidentifying numbers.

	Focus group 1 (in person), participant identifier	Focus group 2 (web-based), participant identifier
Students	1-6	7-9
Preceptors	1-4	5 and 6
Educators	1 and 2	3 and 4

An overall finding of this study is that technology-supported RG meetings led to a better-structured active learning method. The VRG design allowed APNSs to spend more time in clinical practice placements and promoted active and effective participation of APNSs and NPs in the meetings. Participating in the VRG meetings increased the perceived competence of APNSs and NPs. The APNSs also perceived an improved learning experience compared with their own expectations. The findings are presented according to overall themes, followed by subthemes.

The results of this study revealed 3 main themes. The first theme reflects the importance of a well-structured learning activity in creating learning opportunities, whereas the second and third themes reflect how APNSs perceived the achievement of the general required learning outcomes and their expected professional competence as APNs in critical care (Table 5).

**Table 5:** The findings categorized into overall themes and subthemes.

Themes	Subthemes
Preparation process encouraging learning	Importance of a defined teaching design Importance of clearly determined roles
Developing intrapersonal and professional skills	Increased learning focus Increased responsibility and commitment Increased leadership skills
Synergy in competence development	Increased collaboration between students and preceptors Improved focus on evidence-based practice in the clinical environment Improved professional interaction skills

### Preparation Process Encouraging Learning

#### Importance of a Defined Teaching Design

The VRG meetings were conducted based on a rigorous guide, which the students evaluated as “very good.” One of the NEs experienced that the design of the VRG meetings led to better focus, which she perceived as an advantage, especially for the students. Several APNSs thought that the VRG meetings were more structured and effective than the FFRG meetings they had previously experienced (Table 1). The APNSs reported that FFRG meetings resulted in a lack of learning focus after half an hour. All interviewed participants (APNSs, NPs, and NEs) agreed that the newly designed structure for RGs based on virtual meetings improved the participants’ learning experience. In addition, all the NPs in both focus group interviews were very positive about the flexibility and scheduled times of the VRG meetings. The virtual meeting enabled the participation of the users (APNSs, NPs, and NEs) independent of their geographic location, and a shorter meeting allowed the APNSs to spend more time in their clinical practice placement. In addition, the NPs experienced gaining academic competence in both the preparation phase with the APNSs and during VRG meetings. Although the NPs perceived that their academic contribution to the VRG meetings was modest, they perceived a high value in their practical experience in clinical practice and their role as preceptors in support of the APNSs.

The NEs and APNSs from both focus group interviews experienced that the VRG stimulated learning, as patient cases were distributed to the participants before each meeting. This new RG meeting structure led to a perceived improvement in the participants’ focus when compared with the previous design. One student said as follows:

I felt that it led to more learning because it was a completely different way of having a reflection group meeting, and what was presented was more evidence based. We were supposed to present the case and the research in such a way that it was easier to discuss it instead of just sharing our own experiences and opinions.

APNS 7

Another student added the following:

So we come into [the meeting] and we have to just start. We have only 45 minutes, and we have a lot to get through during that time. It became much more academic [with virtual meetings] than when we met face to face; then, it was more like, “Hi, how are you doing?,” and then you maybe lose 10 to 15 minutes talking about what’s been going on and that we haven’t seen each other. We can log in and talk together before the meeting starts if we want to have a chat.

APNS 9

#### Importance of Clearly Determined Roles

An NE pointed out that APNSs were supposed to be on a clinical practicum to learn and that it was important that APNSs, NPs, and NEs understood their roles. One student emphasized the importance of the design in clarifying roles:

Each week, I felt that my role as a student was emphasised; it was easier for me to say, “I’m here to learn new things and to find good learning situations.”

APNS 6

Another student added:

It is important for us that the NP knows what we’re up against, so I think that a good thing about the meetings is that the NPs who have taken part in the VRG meetings know a little bit more about it.

APNS 3

One of the NPs said:

I have understood that the student is the one who takes responsibility, and the preceptor gets involved when the article is found and gets involved with the discussion that takes place before the VRG meeting.

NP 1

Overall, the NPs perceived that they helped as much as they could during the RG meetings. One of the preceptors experienced that everybody had a role to play and that she learned a lot by listening to the respondent giving feedback. One of the educators pointed out:

It is important to involve the clinical preceptor, because it benefits both the student and the preceptor.

NE1

### Developing Intrapersonal and Professional Skills

#### Increased Learning Focus

The students expressed that they spent more time preparing for the VRG meetings than for FFRG meetings. Accordingly, they perceived that they had developed better skills in finding research articles and presenting patient cases. When preparing for the VRG meetings, the APNSs experienced increased learning, as they studied their fellow student’s patient case, demonstrating more committed to being prepared for VRG meetings than for FFRG meetings. They perceived that preparing for the VRG meetings and participating in the patient case discussions stimulated their critical thinking. One of the NPs expressed surprise at the APNSs’ skills:

They [the students] knew the literature well; I had also prepared in advance. The good thing about having these VRG meetings is that they require more preparation of participants when compared with FFRG meetings. Now you have the chance to go a bit more in depth, you can spend more time at the ward, discuss with the NE, discuss with others in your surroundings and other fellow students on the subject, and it means that you maybe develop more insights into the studied subject than before.

NP 2

The VRG meeting was perceived to be evidence based, and the students experienced that each meeting they participated in gradually improved their skills in reading research articles and searching the database. One student said:

I felt that I learned much more than before, because it was a very structured design. I also felt that all the students were well prepared each time. We discussed only the articles that we presented, in addition to which there were different topics each time, not like earlier, where all the students presented the same subject [case], and there was a lot of repetition [before], I felt.

APNS 1

#### Increased Responsibility and Commitment

There was a general agreement among the participants in the focus group interviews that the students participated more in the discussions during the VRG meetings than in the FFRG meetings. The VRG meeting guide stated that all students had to participate actively during the meeting. One student felt that the discussion part of the VRG was uncomfortable, because all the students were to speak in turn, and no student was allowed not to participate in the discussion. However, many other focus group participants experienced the discussion section of the VRG as positive, leading to an increased perception of achieved learning outcomes. It had been easier for the APNSs not to participate in the discussion during FFRG meetings, which negatively affected their learning experience:

You didn’t have to say anything unless you were asked a question. In the virtual meeting, everyone was required to take part, everyone had to prepare, and I think it was good for us as students that you had to take more responsibility in that setting.

APNS 3

The NEs felt that they had a more passive role, as the responsible students and their peers were charged while continuing the discussion. In the VRG meeting, the NEs were able to sit and take notes, which they could then use to provide a summary at the end, which they could not do in the FFRG meetings.

Using a strict guide was also perceived as useful by students who did not like to speak up and therefore would become passive in the previous RG meeting design. One student pointed out:

In the meetings we had on campus, I have noticed that it’s often the same people who take part in the discussions; it’s the same people who speak up, the same who take part in the follow-up discussions, and there are always some who don’t take part. And I think it becomes even more obvious when you are sitting at a screen in a Zoom meeting.

APNS 7

#### Increased Leadership Skills

Some students found it challenging to lead the VRG meetings, feeling that they were outside their comfort zone. The APNSs described diverse experiences related to leading meetings, but they all agreed that it was nerve racking:

The experience of leading a meeting was a bit scary to start with, but I think it would have been just as scary or exciting if I had been in a physical meeting; being on Zoom didn’t make it scarier. Physical meetings could have been scarier. It went very well altogether.

APNS 8

A few students felt it was difficult to encourage their fellow students to discuss the case:

It is scary being the meeting leader for a whole 45 minutes, which none of us have done before. You have to welcome everybody, present a case, then present your research article; then, when you are finished—then—what do you think, and what do you think? You have to carry on the whole time. So it’s not as simple as someone here said earlier, that you can just talk like you do in a normal discussion, but you have to “name drop” like I tend to do; if nobody talks, then I just sort of “name drop” [laughs] so it isn’t just silence. So, yes, it was a bit, being the leader, that was scary but also very educational.

APNS 5

Both APNSs and NEs felt that the VRG meetings were suitable preparations for the role of an APN. For APNSs, it was meaningful to choose a professional topic and discuss it. Some of the APNSs took part in several meetings before they assumed the role of a responsible student:

I managed to prepare myself and learn from the others before I had to do it myself in the end, so I think it [leading a meeting] went OK.

APNS 4

Several of the students experienced enhanced leadership skills by participating in the VRG meetings:

I think we were good at keeping the VRG meeting going. We learned from having to take turns to speak, to include everyone and to ensure that everyone got to say something about their own thoughts and experiences.

APNS 8

The APNSs felt supported by their NPs when performing the leader role:

The students managed to pass on the baton to the other students without it seeming embarrassing; it went quite well. And my role was really, I felt, to support my student through the meeting.

NP6

One student summarized the significance of developing competence in professional leadership:

I felt that the biggest advantage was that we learned to lead a meeting, how to steer it and how to argue. Yes, we are going to become intensive care nurses, but we are also going to become APNs, who will have a slightly different role, so it helped me to see that we will need to be able to lead that type of meeting in a work environment, to be able to take up problems out in the field, try to make changes or show something new; this was a good way of practicing that.

APNS 3

The NEs assumed that the VRG meeting was well structured and promoted APNSs’ development of leadership skills:

Each student practiced leading the professional discussions, and the discussions became very good, and everyone was well prepared.

NE3

### Synergy in Competence Development

#### Increased Collaboration Between Students and Preceptors

The students experienced the NPs’ participation in the VRG meetings as positive. They pointed out that preceptors could not participate in the FFRG meetings because they were unable to leave the ward. The collaboration between NPs and APNSs in the VRG meetings was also seen as positive:

When it came to finding a research article, we had a lot of good conversations about what we wanted to discuss together; we went through several different subjects and found in the end a case that we both found interesting.

APNS 5

In the previous RG design, such cooperation was not possible. Another APNS said:

We discussed a case before the VRG meeting and discussed the results we had found in the article I had chosen. So it was more than what I previously experienced in clinical practicum, where I hadn’t even mentioned the choice of a research article to my preceptor.

APNS 7

#### Improved Focus on Evidence-Based Practice in the Clinical Environment

The NPs experienced that collaborating with the APNSs in the VRG meetings led them to be updated with new knowledge from research publications. Usually, the NPs felt it was challenging to remain up to date on science in their research field because of their hectic, practically orientated daily work. Cooperation between NPs and APNSs stimulated their engagement in evidence-based knowledge:

There is something about the knock-on effects, which are also great when you go about your daily tasks and don’t have much time for additional work as well.

NP1

I think it is important. You get insights into what the students need to learn. You can update yourself, and, as [NP 1] says, there isn’t much time normally to find the newest research, so I think I learned a lot by being a preceptor.

NP2

The NPs also mentioned that the organizational structure of the VRG meetings led to a great deal of involvement, academic interest, and discussions on various topics:

Both I and the student, along with several others, also discussed a difficult case before the student presented it [at the VRG meeting]. We talked about the case afterwards, because there were many who thought that the case was difficult, and, in a way, it became a learning situation for the students but also for us on the ward. So, the VRG meeting was actually a useful method, and there were more people who got something useful out of it. First, the student wrote down the case. I got it as an email, so I could read it on my own time and think about it, and then we all spoke about it on the ward. I think it was a good way of doing it.

NP 5

#### Improved Professional Interaction Skills

For the VRG meetings, the APNSs were instructed to share documents with one another before the meetings, which they perceived as useful. In each meeting, one of the APNSs assumed the role of respondent and had the task of giving the responsible student critical feedback on the chosen research article. The experience of receiving feedback from a fellow student was described as follows:

You get feedback [from the respondent] on how you have appraised the article, and, for me, it was informative and something I can take with me when I am finished. Because you know that you will also use this knowledge later in working life.

APNS 9

One of the NPs felt that the VRG meetings were perfect for cooperation with the APNSs, saying that even though it could be perceived as stressful to read a research article on a busy working day, the preparations for the VRG meetings energized them and helped them give more guidance to the APNSs. The preceptors experienced closer cooperation with the students, as they had a specific task:

You have it in the back of your mind all the time that you have to find a discussion topic together, so there are more professional discussions and learning situations that arise.

NP3

## Discussion

### Principal Findings

The primary findings of this study pertain to the perceived benefits of a structured, active learning approach supported by technology, namely, VRG meetings. When the teaching method is well structured, it generates positive consequences, as shown in our results. The VRG meeting design inspired well-prepared participants because of their well-defined roles and responsibilities, and the APNSs perceived increased competencies related to intrapersonal and professional skills. The VRG meetings also led to increased synergy and collaboration between APNS, NPs, and NEs and, consequently, to perceived enhanced APNS and NP competence.

### The Participants’ Experiences of VRG Meetings

To participate in the VRG meetings, the APNSs had to be prepared, which stimulated their responsibility and commitment to learning. Furthermore, they found that VRG meetings were more effective and focused more on evidence-based knowledge than FFRG meetings. Each week, some APNSs felt that their role as a student was recognized by the NPs, who perceived the APNSs to be more prepared for the meetings and took more initiative and responsibility for diverse learning activities in their clinical practice placement. Providing APNSs with structured, active learning has been found to enable their reflective process and improve their professional practice, and consequently, patient outcomes [12].

The VRG meeting Is a pedagogical method that, in line with Vaz de Carvalho and Bauters [20], fosters active involvement of students in their learning process. According to Agarwal and Kaushik [21], web-based teaching methods should be a part of postgraduate training if they are relevant to students’ learning needs in their clinical practice. Using Zoom as a technological tool in the VRG meetings better established the APNSs’ learning process, as they had to be prepared and could not hide behind others. The use of supportive technology to ensure an active learning process is in line with a recent study by Nes et al [22]. Learning is an active process that requires motivation and engagement from all students, so these elements must be considered when a specific discipline, course, or program aims to guide students toward achieving the required learning outcomes [23]. Higher education programs must be designed to accommodate a new generation of technological learning tools that promote learners’ autonomy, collaboration, and critical analytical ability to foster the active construction of complex knowledge and skills [12,24]. Active learning occurs in interactions between individuals, such as fellow students, who share experiences and knowledge with one another [25]. Our study showed that VRG meetings actively engaged APNSs in the learning process, which is an important finding, as active engagement is crucial in collaborative learning according to Zhang and Cui [24].

The APNSs and NPs who participated in the VRG meetings experienced stimulated critical reflection based on patient cases and available evidence in research articles. APNSs require critical reflection to turn their experiences into learning, for which a structured teaching approach, as implemented in this study is recommended [26]. Critical reflection has also been associated with using analytical cognition in students’ development of problem-solving skills [27]. APNSs need to apply their knowledge to manage complex decision-making in an intensive critical care context. To make the right decision in complex situations requires that APNSs in critical care exercise critical reflection at a high level of analytical cognition because as the Hammond [28] theory of cognition contends, a high level of intuitive cognition may inspire poor decisions. Hammond [28] cognitive continuum theory describes the levels of analytical and intuitive cognition in task management, with task properties varying from poorly to well structured [29]. Analytical cognition is associated with cognitive control, slow data processing, and conscious awareness and confidence, which are often induced when managing a well-structured task. However, ill-structured tasks such as complex patient situations in critical care often induce intuitive cognition, which involves less cognitive control, less conscious awareness, and low confidence [28,29]. By attending the VRG meetings, the students turned their experiences into learning using critical reflection with analytical cognition, discussing difficult cases, sharing knowledge, and reaching a deeper level of assessment and a higher level of cognition, as recommended by Miraglia and Asselin [12] and Scheel and Bydam [14].

### The Main Perceived Improved APNS Competencies Resulting From VRG Meetings

Our results indicate that participating in VRG meetings was experienced as a good preparation for the role of an APN, primarily with regard to the development of intrapersonal and professional skills, which embrace a nurse’s capability to understand, deal with emotions, and practice self-discipline [30]. In this study, the APNSs dealt with emotions (feeling outside their comfort zone) by leading and actively participating in VRG meeting discussions. In addition, the meetings contributed to greater responsibility and commitment of the APNSs in terms of preparation and participation when compared with FFRG meetings. In the professional role of an APN in critical care, the meaning of competence is feeling sufficiently safe and secure to efficiently manage decision-making in life-threatening patient situations [31]. Our findings clearly show that the APNSs perceived the VRG meetings as meaningful, and they reported that choosing a patient case and relevant research study, leading the meeting, and being required to argue increased their self-discipline and self-confidence. Furthermore, by participating in the VRG meetings, the APNSs gradually gained the confidence in presenting their point of view, which contributed to the development of an autonomous role and advanced knowledge in clinical decision-making in critical care, as expected from an APN [32,33]. Implementing VRG meetings in the clinical practice of master’s education programs may positively enhance APNSs’ personality traits, which affect their conscientiousness and openness to experience in developing their competence and are important factors in nursing education in critical care [31].

Another important finding of this study was students’ ability to develop their leadership skills, a core competency required in the APN role [6,32]. Essential leadership skills in APNs include competence in self-awareness, self-management, social awareness, and relationship management [6]. In this study, the responsible student ensured that everyone attending the meeting had the opportunity to express their thoughts and experiences. These discussions became very positive, increasing the synergy and competence development among the APNSs. The VRG meetings also influenced the clinical practice environments of the clinical placements at both the individual and organizational levels. At the individual level, reflection leads to enhanced knowledge and transforms the assumptions. At the organizational level, reflection empowers nurses to explore concerns and make changes [12]. The results of our study are in line with those of Ljungbeck et al [32], who described leadership skills as an important competence for APNSs in critical care. The results of this study regarding APNSs’ perceived achievement of leadership skills may be transferable to the clinical practice context, potentially enabling them to develop professional leadership skills in the ward.

### Strengths and Limitations

This innovative study used technology to improve the teaching approach (RG meetings) routinely used in clinical practice for nursing education. The data were collected from all parties (APNSs, NPs, and NEs) involved in clinical practice education, increasing the trustworthiness of the intervention evaluation. Data were collected from 2 different groups at different stages of the APN education program. Moreover, the developed VRG meeting can easily be adapted to several educational programs and to various levels of professional education.

As a limitation, we experienced a slight drop out of possible informants in the second focus group interview. One reason for this may be that we invited a larger number of students, as VRG meetings were implemented in a greater number of CCUs (Table 3). Another reason may be that the interview was in a web-based format because of the COVID-19 pandemic (although we found this perplexing, as the informants were used to attending virtual meetings). However, the low number of participants in the second interview confirmed that the informants felt no pressure to participate in this study, which was positive. Furthermore, VRG meetings depend on appropriate and functional technical tools, and participants must have access to devices, such as computers, tablets, or smartphones.

### Conclusions

The participants perceived the VRG meeting—a structured, active learning approach supported by technology—as being more effective than FFRG meetings on campus in developing APNSs’ required competence in critical care. The VRG meeting was also perceived as an improved approach for solving several challenges previously experienced in FFRG meetings. On the basis of participants’ experiences, we conclude that VRG meetings contribute to increasing APNSs’ competence, specifically by preparing them to exercise complex decision-making with a higher level of analytical cognition in a clinical context. VRG meetings may also inspire professional discussions in the ward, increasing professional interaction.

## Abbreviations

APN: advanced practice nurse
APNS: advanced practice nursing student
CCU: critical care unit
FFRG: face-to-face reflection group
LDUC: Lovisenberg Diaconal University College
NE: nurse educator
NP: nurse preceptor
RG: reflection group
VRG: virtual reflection group

## Data Availability

The data sets generated and analyzed during this study are available from the corresponding author on reasonable request.
